# Divergent roles of the Hippo pathway in the pathogenesis of idiopathic pulmonary fibrosis: tissue homeostasis and fibrosis

**DOI:** 10.1186/s41232-023-00295-1

**Published:** 2023-09-21

**Authors:** Ryusuke Kizawa, Jun Araya, Yu Fujita

**Affiliations:** 1 Division of Respiratory Diseases, Department of Internal Medicine, The Jikei University School of Medicine, 3-25-8 Nishi-Shimbashi, Minato-Ku, Tokyo, 105-8461 Japan; 2 Division of Next-Generation Drug Development, Research Center for Medical Sciences, The Jikei University School of Medicine, Tokyo, Japan

**Keywords:** Idiopathic pulmonary fibrosis, The Hippo pathway, Epithelial cells, Fibroblasts, Tissue homeostasis, Mechanosignaling

## Abstract

Idiopathic pulmonary fibrosis (IPF) is a progressive aging-related lung disease with a poor prognosis. Despite extensive research, the cause of IPF remains largely unknown and treatment strategies are limited. Proposed mechanisms of the pathogenesis of IPF are a combination of excessive accumulation of the extracellular matrix and dysfunctional lung tissue regeneration. Epithelial cell dysfunction, in addition to fibroblast activation, is considered a key process in the progression of IPF. Epithelial cells normally maintain homeostasis of the lung tissue through regulated proliferation, differentiation, cell death, and cellular senescence. However, various stresses can cause repetitive damage to lung epithelial cells, leading to dysfunctional regeneration and acquisition of profibrotic functions. The Hippo pathway is a central signaling pathway that maintains tissue homeostasis and plays an essential role in fundamental biological processes. Dysregulation of the Hippo pathway has been implicated in various diseases, including IPF. However, the role of the Hippo pathway in the pathogenesis of IPF remains unclear, particularly given the pathway’s opposing effects on the 2 key pathogenic mechanisms of IPF: epithelial cell dysfunction and fibroblast activation. A deeper understanding of the relationship between the Hippo pathway and the pathogenesis of IPF will pave the way for novel Hippo-targeted therapies.

**Supplementary Information:**

The online version contains supplementary material available at 10.1186/s41232-023-00295-1.

## Background

Idiopathic pulmonary fibrosis (IPF) is a progressive aging-related lung disease of unknown etiology. Characteristics of IPF include excessive deposition of the extracellular matrix (ECM) and tissue remodeling of the lung, leading to respiratory failure [1, 2]. The prognosis of IPF is poor, with a reported median survival time among Americans 65 years or older of 3.8 years [3]. For treating IPF only 2 antifibrotic agents—pirfenidone and nintedanib—are now available [4, 5]. Although these agents have been shown to significantly reduce the mortality rate of IPF, more than 30% of patients still die within 2 years after diagnosis [6]. Moreover, antifibrotic agents only slow the progression and are not able to reverse the fibrosis. Thus, further understanding the pathogenic mechanisms of IPF and developing a novel therapy are urgent tasks.

The pathogenesis of IPF has 2 critical components: the activation of fibroblasts and the dysfunction of lung epithelial cells [2]. Fibroblast activation is a key process for the accumulation of ECM. Fibroblasts are usually in a quiescent state, but in response to developmental cues or tissue injury, they become activated and differentiate into myofibroblasts [7]. Myofibroblasts are characterized by increased contractility and expression of α smooth muscle actin (α-SMA). The myofibroblasts produce ECM and modulate the functions of immune cells to perform tissue repair. However, persistent activation of fibroblasts due to repetitive tissue damage leads to fibrosis. The mechanism of fibroblast activation is complex and involves many factors. Proposed critical causes of fibroblast activation have included mechanosignaling, crosstalk between fibroblasts and other cells, the innate immune system, and cytokines, such as transforming growth factor (TGF)-β [8]. In addition, an important mechanism for fibrosis is the convergence of the Hippo, TGF-β, and Wnt signaling pathways [9]. On the other hand, repetitive damage in the lung is believed to exhaust the regenerative capacity of epithelial cells, leading to the dysregulation of the lung tissue homeostasis [10]. Recent studies with single-cell RNA sequencing (scRNA-seq) have revealed unique and previously undescribed cellular populations in IPF lungs. In particular, epithelial cells with dysregulated differentiation are profibrotic, suggesting the burden of the dysfunctional epithelial cells on the pathogenesis of IPF [11–19].

The Hippo pathway is a central signaling pathway for regulating the fundamental functions of epithelial cells, such as proliferation, differentiation, regeneration, and regulated cell death, and contributes to the maintenance of tissue homeostasis [20]. Largely dependent on the Hippo pathway is the maintenance of cellular functions of lung epithelial cells. An additional unique feature of the Hippo pathway is mechanosignal transduction [21]. Thus, the Hippo pathway likely plays a pivotal role in the pathogenesis of IPF. In this review, we discuss the diverse roles of the Hippo pathway in IPF. We highlight the seemingly opposing functions of the Hippo pathway in epithelial cell dysfunction and fibroblast activation. Furthermore, we provide therapeutic strategies for IPF by comprehensively presenting potential drug candidates that modulate the Hippo signaling, and propose cell type- and stage-specific targeting of the Hippo pathway in IPF pathogenesis.

## Main text

### Overview of the Hippo pathway

**Fig. 1:**
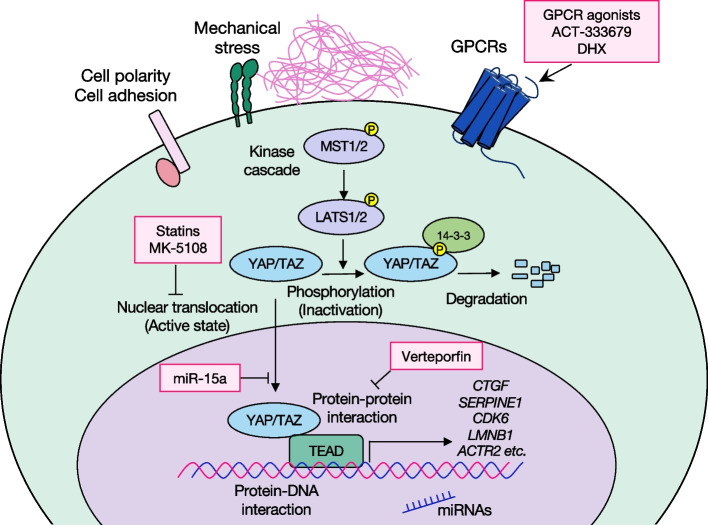
The Hippo signaling pathway and therapeutic targets. The Hippo signaling pathway consists of the kinase cascade component in the cytoplasm and the YAP/TAZ-TEAD transcription component in the nucleus. The pathway is regulated by numerous stimuli, including mechanical stress, GPCRs, cell polarity, and cell adhesion. When the Hippo pathway is “on,” the kinase cascade transduces the stimuli, ending with YAP/TAZ phosphorylation. Phosphorylated YAP/TAZ bind with 14–3-3 and undergo ubiquitin-proteasomal degradation. In the Hippo “off” condition, YAP/TAZ are activated in a dephosphorylated state and are translocated into the nucleus to modulate transcription. The possible targets of the Hippo pathway are: i) upstream regulators of YAP/TAZ, ii) YAP/TAZ nuclear translocation, iii) protein–protein interaction between YAP/TAZ and TEAD, iv) protein-DNA interaction between YAP/TAZ-TEAD and target DNA, and v) transcription and expression of the target genes. Candidate compounds shown in the diagram are ACT-333679, DHX, statins, MK-5108, verteporfin, and miR-15a. The GPCR ligands are ACT-333679, which is a partial agonist of IP receptor, and DHX, which is a full agonist of DRD1. The statins are HMG-CoA reductase inhibitors and MK-5108, which is a selective inhibitor for Aurora kinase A; they indirectly inhibit the nuclear translocation of YAP. Verteporfin blocks the protein–protein interaction of YAP/TAZ and TEAD, and MiR-15a decreases YAP expression

The Hippo pathway is an evolutionarily conserved signaling pathway, initially identified in *Drosophila* by screening for tumor suppressor genes [22]. Genetic inactivation of this pathway results in tissue overgrowth, which suggested its key role in controlling organ size [22]. Recent studies have shown the Hippo pathway to be involved in fundamental biological processes, including proliferation, differentiation, regeneration, development, and immune modulation [20]. The Hippo pathway has unique upstream signals, such as mechanical signals, cell polarity, cell adhesion, G protein–coupled receptor (GPCR) signaling, and other soluble factors [21] (Fig. 1). The key components of the Hippo pathway are mammalian sterile 20 (STE20)-like protein kinase (MST)1/2; large tumor suppressor (LATS)1/2; Yes-associated protein (YAP); transcriptional coactivator with PDZ (postsynaptic density protein 95 [PSD-95], *Drosophila* disc large tumor suppressor [DlgA], and zona occludens 1 [ZO-1])–binding motif (TAZ); and a transcriptional enhancer associate domain (TEAD) family member. Both MST1/2 and LATS1/2 are serine-threonine kinases forming the Hippo kinase cascade. The cascade ends with LATS1/2 directly phosphorylating and inactivating YAP and TAZ (YAP/TAZ) [23]. Both YAP/TAZ are highly related transcription coactivators that act as effectors of the Hippo pathway [23]. In addition, YAP/TAZ shuttle between cytoplasm and nucleus depending on the phosphorylation state so that they transduce the extracellular stimuli into the transcriptional cues. When phosphorylated, YAP/TAZ bind to 14–3-3 and are sequestered in the cytoplasm, leading to their ubiquitin-mediated proteasomal degradation [23]. Conversely, dephosphorylated YAP/TAZ undergo nuclear translocation and actively regulate transcription via binding to transcription factor TEAD in the nucleus [23].

Dysregulation of the Hippo pathway is believed to cause various diseases. Perhaps the most notable consequence of dysregulation is cancer progression because the pathway is an important regulator of cell proliferation and appropriate tissue growth. Indeed, in many types of cancer, overactivation of YAP/TAZ is observed and leads to aberrant cell growth evading contact inhibition, increased resistance to apoptosis, epithelial-mesenchymal transition, and the gain of cancer stem cell–like properties [24]. Interestingly and paradoxically, emerging evidence suggests that YAP has tumor-suppressing functions and that LATS1/2 have tumor-promoting functions via interactions with other signaling pathways or modulating cancer microenvironment [24, 25]. Further research is needed to understand the close and complex relationship of the Hippo pathway and the pathogenesis of cancer.

Another important result of Hippo pathway dysregulation is fibrosis. The Hippo pathway is essential for mechanotransduction, which makes the pathway reasonable to have a significant effect on organ fibrosis or tissue stiffening diseases. Many studies have confirmed that the Hippo pathway is involved in fibroblast activation, the direct pathogenesis of fibrosis, in various organs, including the lung, liver, kidney, heart, and skin [26]. Although most studies suggest that the Hippo pathway negatively regulates fibrosis, the pathway can also play a profibrotic role [26, 27]. For example, in the liver, YAP activation is believed to reduce inflammation by protecting hepatocytes and to prevent the activation of hepatic stellate cells [27]. However, owing to the reciprocal interactions between fibroblasts and other cell types, such as epithelial cells and immune cells, fibrotic disease is difficult to fully understand [8]. Understanding the involvement of the Hippo pathway in such intercellular communications should help us clarify in detail the pathogenesis of fibrosis.

The expression profile of the components of the Hippo pathway in the lung has been investigated. In adult mouse lungs, YAP mRNA expression level is much higher in basal cells than in ciliated cells or secretory cells [28]. YAP is normally localized in the nucleus of the basal stem cells [28]. During rodent lung development, expression of epithelial YAP and TAZ are required at the early stage and the later stage respectively, and they play roles in bronchial morphogenesis by regulating *SHH* expression [29]. In normal human lungs, with immunohistochemistry, YAP expression has been shown in the nucleus of alveolar type (AT) 2 cells [30]. Conversely, LATS is rarely detected in alveolar cells and is expressed in basal cells of the bronchiole [30]. On the other hand, in the IPF lungs, YAP expression is increased in the nucleus of epithelial cells, which suggests the potential roles of the Hippo pathway in IPF pathogenesis [31].

### Function of lung epithelial cells and the Hippo pathway

The pathogenesis of IPF is considered a combination of epithelial cell dysfunction and fibroblast activation. Although fibroblast activation leading to excessive accumulation of ECM is a clear and direct cause of IPF, dysfunctional epithelial cells play a critical role in the initiation and progression of IPF. In normal lungs, tissue homeostasis is maintained by the function of epithelial cells, including proliferation, differentiation, regeneration, cell death, and cellular senescence. These cellular functions are disturbed owing to repetitive cellular damage resulting from both intrinsic and extrinsic stresses, such as loss of proteostasis, endoplasmic reticulum stress, mitochondrial dysfunction, inflammatory cytokines, infection, and cigarette smoke. Dysfunctional epithelial cells are expected to be therapeutic targets for IPF, and their characteristics and pathogenic functions have been intensely investigated.

Each cell type in the lungs of human patients with IPF has recently had, with the scRNA-seq technique, its transcriptional profiles revealed, and previously undescribed populations of epithelial cells have emerged as key players in the pathogenesis of IPF [11–19]. Several different studies have identified similar populations of pathogenic epithelial cells that co-express markers of both basal epithelial cells and mesenchymal cells, which are localized adjacent to the fibrotic foci [17, 18]. That these pathogenic epithelial cells express mesenchymal cell markers but lack some typical airway basal cell markers, such as keratin 5 (KRT5), indicates their indeterminate and defective functions [17, 18]. The pathogenic epithelial cells, called “aberrant basaloid cells”, are characterized by high expression of 2 established IPF markers—matrix metallopeptidase 7 (MMP7) and α_V_β_6_ integrin—which suggests their profibrotic functions [17, 18]. In addition, these populations of epithelial cells show increased expression of replicative senescence-associated genes, including *CDKN1A*, *CDKN2A*, and *MDM2* [17, 18]. Another IPF lung–derived basal cell population that has been identified has overlapping features of “aberrant basaloid cells” but is definitely different from them [19]. These cells exhibit de-differentiation and promote proliferation and ECM production of lung fibroblasts. Although the origin and the causal factors of these basaloid cell populations remain unclear, their unique mesenchymal features and cellular senescent phenotype suggest that they emerge in response to disturbance of lung tissue homeostasis. Involvement of epithelial-mesenchymal transition (EMT) leading to fibroblast accumulation in the pathology of IPF is controversial, but dysregulated EMT has been considered to represent dysfunctional tissue regeneration and seemingly contribute to the pathogenesis of IPF [32–35]. The basaloid cells with mesenchymal features might be the result of such dysregulated EMT. Notably, the basaloid cell populations are found almost exclusively in fibrotic lungs and share profibrotic functions, indicating their burden on the pathogenesis of IPF.

In addition to basaloid cells, certain injury-induced populations of alveolar epithelial cells have been reported to appear in IPF lungs. Among them, AT2 cells isolated from IPF lungs have high expressions of cellular senescence-related genes and contribute to progressive pulmonary fibrosis [12, 16]. Moreover, in the regeneration process of murine lung subsequent to injury induced by lipopolysaccharide or bleomycin, lung epithelial cells exhibit an intermediate state during the transition from AT2 cells to AT1 cells, which have been identified independently by 3 research groups [14, 15, 36]. Lung epithelial cells in an intermediate state have been shown to accumulate in IPF lungs and share cellular senescent features, suggesting dysfunctional alveolar epithelial regeneration contributing to the pathogenesis of IPF [14, 15, 36]. The newly identified clusters of dysfunctional epithelial cells have been shown to be key players in the pathogenesis of IPF. These cells are believed to appear in response to lung tissue injury. Of note, in intermediate alveolar epithelial cells, YAP/TAZ are activated, although their role is unclear [14, 15]. Because the other enriched pathways, namely tumor protein p53 (TP53), nuclear factor (NF)-κB, TGF-β, and hypoxia-inducible factor 1 (HIF1), are related to lung tissue regeneration, YAP/TAZ might have been activated to exert regenerative function, but their potential efficacy is hindered by dysregulated differentiation [14]. Although the pathological significance of the intermediate cells remains unclear, the Hippo pathway is believed to play a crucial role in maintaining lung tissue homeostasis.

**Fig. 2:**
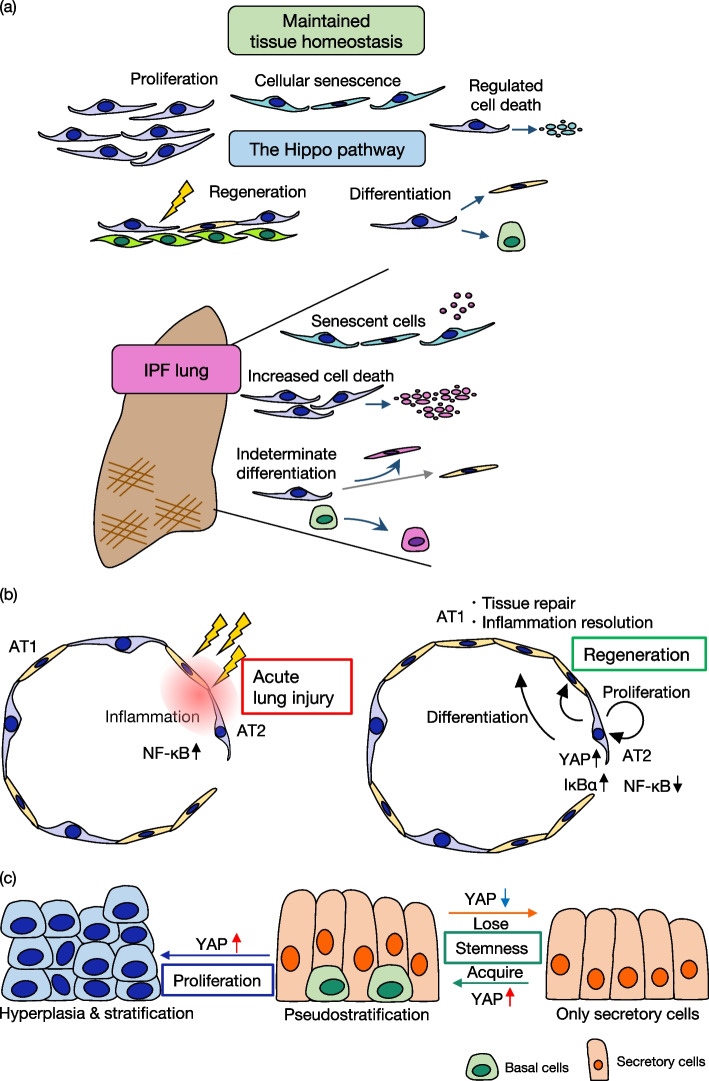
Diverse roles of the Hippo pathway in epithelial cells and progenitor cells. **a** The Hippo pathway is involved in fundamental cellular functions, such as proliferation, differentiation, regeneration, cellular senescence, and regulated cell death. The appropriate regulation of the Hippo pathway in lung epithelial cells is essential for the maintenance of tissue homeostasis. Observed in IPF lungs are dysfunctional epithelial cells. In particular, epithelial cells with impaired differentiation, leading to features of both basal and mesenchymal cells, have been revealed as pathogenic epithelial populations. Furthermore, increased senescent cells and cells undergoing cell death due to repetitive cellular stresses play an important role in the progression of IPF. **b** Alveoli are lined by AT1 and AT2 cells. In acute lung injury, NF-κB is upregulated in the AT2 cells, and inflammation occurs. In response to the damage, YAP is activated in AT2 cells and promotes their proliferation. Furthermore, YAP induces IκBα expression in AT2 cells for resolution of the inflammation. In addition, upregulated YAP causes AT2 cells to differentiate into AT1 cells to promote tissue repair. **c** In the airway, basal progenitor cells and secretory cells comprise the pseudostratified epithelial architecture. Both types of cells transit reciprocally between each other, depending on YAP expression. When YAP is downregulated in basal progenitor cells, which then differentiate into secretory cells. In contrast, increased YAP expression in the differentiated cells causes them to dedifferentiate and acquire stemness. On the other hand, YAP overexpression in basal progenitor cells leads to their proliferation and stratification. With these findings, YAP can be considered a key factor for regulating tissue regeneration after injury by balancing quiescent and proliferative states

To maintain lung tissue, appropriate differentiation, cellular proliferation, and regeneration are essential. These cellular functions are closely related to each other and are regulated by the Hippo pathway **(**Fig. 2**)**. The transitions of progenitor cells and differentiated secretory cells to each other are regulated by the YAP expression level. The loss of YAP in basal stem cells causes them to differentiate into secretory cells [28]. Conversely, YAP overexpression in stem cells promotes their proliferation and inhibits terminal differentiation, which lead to dysregulated epithelial hyperplasia and stratification. On the other hand, excessive YAP expression causes secretory cells to acquire stem cell-like properties. In contrast, suppression of YAP inhibits the de-differentiation of secretory cells into basal stem cells. The relationship between club cells and goblet cells in the conducting airways is similar to that of basal cells and secretory cells. Club cells function as stem cells capable of self-renewal and differentiation, but extrinsic stimulations such as cigarette smoke, allergens, and infection can induce them to differentiate into goblet cells [37]. YAP/TAZ restrict goblet cell differentiation by suppressing the *SPDEF* in the homeostatic state, and YAP/TAZ deletion can lead to goblet cell metaplasia, which is a hallmark of many chronic respiratory diseases including IPF [37, 38]. The regulation of differentiation by YAP suggests its crucial role in maintaining lung tissue homeostasis.

During normal turnover and tissue regeneration, important processes are the proliferation of AT2 cells and their differentiation into AT1 cells [39]. Interestingly, in the regeneration process of acute lung injury (ALI), inflammatory cells transiently increase as AT1 cells are lost and fibrotic lesions emerge, after which inflammatory cells resolve and AT1 cells recover [40]. Recent studies have shown that after ALI occurs, YAP expression increases in AT2 cells [40, 41]. Increased nuclear YAP causes AT2 cells to proliferate and differentiate into AT1 cells for tissue repair and the resolution of inflammation [40]. Especially in regard to the resolution of inflammation, YAP functions through upregulating NF-κB inhibitor α, leading to NF-κB–mediated inflammation being inhibited [40]. Furthermore, in the IPF lungs, YAP expression is increased in the nucleus of epithelial cells and has been shown to promote epithelial cell migration and proliferation, which suggests its contribution to the regenerative function of epithelial cells [31]. Additionally, YAP expression in lung endothelial cells has been shown to elevate during regeneration after unilateral pneumonectomy, and YAP promotes the proliferation of both endothelial cells and epithelial cells [42]. These findings indicate that the Hippo pathway is important for establishing a level of equality between tissue damage and regeneration. In particular, YAP activity in epithelial cells is essential for tissue regenerative processes.

Cellular senescence is an important biological process characterized by the loss of proliferative capacity. Cellular senescence is induced in response to tissue injury caused by repetitive DNA damage. Accumulation of senescent epithelial cells and myofibroblasts is a key mechanism of the pathogenesis of IPF [16, 43, 44]. The senescent cells exert profibrotic functions via the senescence-associated secretory phenotype, leading to fibroblasts being activated in paracrine and autocrine manners [43, 44]. Accumulating evidence shows that YAP/TAZ negatively regulate cellular senescence in several diseases; however, the regulation of cellular senescence by YAP/TAZ in IPF has rarely been reported [45–50]. Because cellular senescence plays a critical role in the pathogenesis of IPF, more must be understood of the effect of the Hippo pathway on cellular senescence in IPF lungs.

As a contradictory counterpart to cellular proliferation, regulated cell death plays a pivotal role in maintaining lung tissue homeostasis. Dysregulation of epithelial cell death led by numerous cellular stresses results in the pathogenesis of IPF [51, 52]. For example, AT2 cells undergo apoptosis so that IPF progresses through DNA damage, telomere shortening, oxidative stress, and mitochondrial dysfunction [53–56]. In addition, necroptosis of alveolar epithelial cells induces the release of damage-associated molecular patterns and thereby triggers the pathogenesis of IPF [57]. Although epithelial cell death regulated by the Hippo pathway has not yet been reported in the pathogenesis of IPF, cell death and the Hippo pathway are closely related and might contribute to the progression of IPF [58, 59].

### Lung fibroblast activation and the hippo pathway

Fibrosis is composed of excessive deposition of ECM, resulting from a dysregulation of tissue repair [8, 60]. Proposed as key mechanisms of organ fibrosis are mechanosignaling; cytokines, such as TGF-β; cellular crosstalk; and an innate immune system [8]. The direct cause of fibrosis is activated fibroblasts. Fibroblasts are normally in a quiescent state, in which they are nonproliferative and maintain the ECM homeostasis [7, 8, 61]. Fibroblasts are activated by repetitive tissue injury, which causes quiescent fibroblasts to differentiate into myofibroblasts [8, 62]. Myofibroblasts are characterized by increased contractility due to α-SMA expression and increased ECM production [7].

The proteins YAP/TAZ are believed to induce fibrosis in concert with TGF-β and Wnt signaling, which share a unique feature of regulating transcription via extracellular/cytosolic signaling and nuclear translocation [9]. Signaling by TGF-β is established as an essential regulator of tissue fibrosis [63]. In the canonical TGF-β signaling pathway, TGF-β binds to its receptor and the signaling domain of the receptor phosphorylates Smad2/3 to form a complex with Smad4 [9]. The activated Smad complex translocates into the nucleus to promote the transcription of genes, such as *PAI1, COL1A1,* and *CCN2*, encoding profibrotic molecules [9]. The activity of the Smad complex is negatively regulated by inhibitory Smad7, which forms a negative feedback loop [9].

Known to regulate fetal development, Wnt has recently been shown to be involved in the pathogenesis of IPF [64, 65]. In the canonical Wnt signaling pathway, Wnt ligands bind to transmembrane receptors—Frizzled (FZD) and low-density lipoprotein receptor-related protein (LRP)—leading to LRP phosphorylation by glycogen synthase kinase (GSK) 3 and casein kinase (CK) 1 in the cytoplasm [9]. The destruction complex, composed of Disheveled (DVL), Axin, adenomatous polyposis coli (APC), GSK3, and CK1, is inhibited to interact with ubiquitin ligase β-transducin repeat-containing protein (β-TrCP). The effector protein β-catenin evades ubiquitination by β-TrCP and subsequent proteasomal degradation and undergoes nuclear translocation to interact with T-cell factor (TCF)/lymphoid enhancer-binding factor-1 (Lef-1) transcription factors and to regulate the transcription of the target genes [9]. Each component of these signaling pathways affects each other by regulating the expression of the ligands, cytoplasm-nucleus shuttling of the effector molecules, and recycling of the transcription factors [9]. For example, regarding the components of the Hippo pathway, YAP binds to Smad7 and enhances the suppressive effect of Smad7 on TGF-β signaling. Conversely, YAP/TAZ also interact with the activated Smad complex, leading to enhanced transcription activity. In addition, YAP/TAZ associate with Axin in the Wnt-off state and are retained in the cytoplasm [66]. In contrast, cytoplasmic YAP/TAZ are required for the destruction complex function of the Wnt pathway [66]. Therefore, Wnt signaling results in both YAP/TAZ and β-catenin nuclear translocation. Furthermore, phosphorylated YAP directly binds to β-catenin to inhibit its nuclear translocation [67].

In the pathogenesis of IPF, the Hippo pathway and TGF-β signaling synergistically cooperate to promote fibrosis. The fibrogenic cooperation of YAP and TGF-β is regulated by GPCR signaling. Three nonspecific GPCR ligands— lysophosphatidic acid (LPA), sphingosine-1-phosphate (S1P), and thrombin—have been shown to activate YAP/TAZ [68, 69]. In cooperation with TGF-β, LPA, S1P, and thrombin synergistically upregulate plasminogen activator inhibitor type 1 (PAI-1) secretion and connective-tissue growth factor (CTGF) expression [70]. The profibrotic response to GPCR activation is mediated by Rho-dependent YAP activation and increased YAP-Smad2 interaction in the nuclei of fibroblasts [70]. The ligand S1P has been shown to be essential for TGF-β–induced YAP activation, leading to the production of mitochondrial reactive oxygen species (mtROS) in human lung fibroblasts [71]. Conversely, a partial agonist of prostacyclin (IP) receptor, a Gα_s_-coupled GPCR, shows antifibrotic activity by inhibiting YAP/TAZ and decreasing YAP-Smad interaction in human primary lung fibroblasts [72]. The convergence of the Hippo pathway and Wnt signaling has not been shown in IPF fibroblasts. However, in IPF epithelial cells, the 2 signaling pathways are activated and YAP is reported to negatively regulate Wnt signaling; these findings suggest that YAP-Wnt interaction is involved in the progression of IPF [31]. The intricate convergence of signaling pathways presents a challenge in developing antifibrotic drugs that specifically target a single signaling pathway. To overcome this obstacle, further studies are needed of protein–protein interactions and transcription modulations in the pathogenesis of IPF.

Mechanosignaling is a major mechanism of fibroblast activation. Mechanical stresses, such as ECM stiffness, and increased cell contractility activate fibroblasts through profibrotic signaling molecules, such as TGF-β and Rho-kinase (ROCK) [73–76]. Typically, once profibrotic signaling has been induced by stiffness, a positive feedback loop is formed, which explains the progression of fibrosis. The Hippo pathway uses mechanosignaling as a distinctive upstream signal. In addition to TGF-β signaling, YAP/TAZ have been demonstrated by recent studies to be mechanoactivated and to modulate the gene expression of lung fibroblasts when grown on a stiffened matrix [75, 77]. Remarkably, the altered cellular features of IPF-derived fibroblasts, including cell morphology, increased proliferation, contractility and fibrotic matrix synthesis, are reversed by silencing YAP/TAZ; this reversal indicates the considerable contribution of YAP/TAZ in the stiffness-induced activation of lung fibroblasts [77]. The protein YAP has been shown to regulate TGF-β signaling depending on the stiffness of tissues [78]. Contractile activation of fibroblasts induced by TGF-β has recently been shown to be cooperatively controlled by YAP/TAZ [79]. Moreover, in fibrotic organs, TGF-β and YAP/TAZ activate NUAK family SNF1-like kinase 1 (NUAK1), leading to further activation of TGF-β/Smad and YAP signaling in fibroblasts [80]. Collectively, TGF-β and YAP/TAZ form a stiffness-related positive feedback loop in a complicated manner. Furthermore, mechanoactivated YAP/TAZ can exert their fibrogenic effect independently of TGF-β signaling. The fibrogenic effect can be elicited by stiffness-dependent and TGF-β–independent expression of PAI-1 [77]. The mechanoactivated YAP/TAZ and PAI-1 reinforce matrix stiffness and form another positive feedback loop, resulting in sustained fibrosis in the pathogenesis of IPF. Unlike other mechanisms, fibroblast activation via mechanosignaling forms this positive feedback loop. The Hippo, TGF-β, and Wnt signaling pathways share these respective negative feedback mechanisms: phosphorylation of YAP/TAZ, inhibitory Smad7, and the destruction complex. Therefore, the mechanosignaling likely plays a central role in the progressive fibrosis of IPF lungs.

### Therapy for targeting the Hippo pathway

Lung fibrosis is closely related to the Hippo pathway, which is likely to be a therapeutic target. Because the pathogenesis of fibrosis consists of both the convergence of various signaling pathways in the fibroblasts and of complex intercellular communications, fibrosis might be difficult to fully control by modulating only a single pathway. However, being strongly involved in maintaining tissue homeostasis, the Hippo pathway is a promising candidate for a therapeutic target of fibrosis. Fibrosis is considered a result of an imbalance of cellular stress and normal tissue regeneration. The Hippo pathway plays a key role in both aberrant fibroblast activation and benign regeneration. A unique characteristic is that the Hippo pathway mediates mechanosignaling.

Treatment targeting the Hippo pathway would certainly have effects on fibrosis. As mentioned earlier, YAP/TAZ signaling has both tissue homeostasis maintenance effects and profibrotic effects, which are apparently conflicting in the causative mechanisms of IPF. Interestingly, the profibrotic effect is at least partly induced by mechanosignaling, and the increased stiffness and YAP/TAZ activation form a positive feedback loop. A reasonable conclusion is that the tissue stiffness-YAP/TAZ feedback loop causes the fibrosis to be sustained and to progress. Therefore, targeting YAP/TAZ can be expected to break the feedback loop and to slow or even stop the fibrosis. To affect the transcriptional regulation by YAP/TAZ, possible therapeutic targets are as follows: upstream molecules that regulate YAP/TAZ activity, activation and nuclear translocation of YAP/TAZ, protein–protein interaction between YAP/TAZ and TEAD, protein-DNA interaction between the YAP/TAZ-TEAD complex and binding DNA, and downstream molecules regulated by YAP/TAZ transcription (Fig. 1). The classical inhibitor of YAP/TAZ is verteporfin. Although verteporfin directly inhibits the YAP-TEAD interaction, its use as a drug is inappropriate owing to nonspecific effects and high insolubility [81]. Developing inhibitors for protein–protein or protein-DNA interaction has been considered impossible. Unlike inhibiting enzymes, pockets for active sites do not exist in the interactions, which makes transcription factors difficult to target. Despite the challenge, accumulating research has made progress in targeting transcription factors in cancers [82]. Similarly, the targeting of transcription factors can be applied to developing drugs to treat fibrosis, especially in YAP/TAZ-TEAD-DNA interactions.

Two compounds, the active metabolite ACT-333679, which is a partial agonist of the IP receptor, and dihydrexidine (DHX), which is a full agonist of dopamine receptor D1 (DRD1), are both GPCR ligands and exert their antifibrotic effects by inhibiting YAP/TAZ nuclear translocation [72, 83]. These compounds were found via the aim of regulating GPCRs expressed in fibroblasts. In addition, screening YAP/TAZ inhibitors with high-throughput technology has achieved significant results. Inhibitors of the profibrotic effects of YAP/TAZ which have been identified include statins, hydroxymethylglutaryl-coenzyme A (HMG-CoA) reductase inhibitors, and MK-5108, a selective inhibitor for Aurora kinase A, [84, 85]. Statins and MK-5108 were screened for small molecules that inhibit the nuclear localization of YAP in primary human lung fibroblasts [84, 85] and are thought to indirectly prevent YAP from nuclear translocation; however, the mechanisms are not fully understood. Because statins and MK-5108 are already available for treating other diseases, their safety is, to some extent, guaranteed. Furthermore, because statins and MK-5108 possibly have different direct targets, combining them might demonstrate synergetic effects on inhibiting YAP activation.

Another possible novel drug modality is microRNAs (miRNAs). Overexpression of miR-15a alleviates fibrosis in a bleomycin-induced rodent model by reducing the expression of YAP [86]. In that study, miR-15a was overexpressed by using adenovirus-associated virus 5 as a vector [86]. An increasing number of studies have shown that extracellular vesicles (EVs) are promising cargoes for miRNAs. A recent study has demonstrated that EVs that have been derived from human bronchial epithelial cells and contain miRNAs inhibit TGF-β–induced activation and senescence of fibroblasts [87]. The antifibrotic effect was due to downregulation of Wnt and inhibition of crosstalk between TGF-β and Wnt signaling pathways [87]. The close relationship of TGF-β, Wnt, and YAP/TAZ signaling pathways suggests that EVs are potential candidates for drugs to modulate YAP/TAZ.

A possible start of fibrosis is the loss of balance between cellular stress and cell-protective factors. Therefore, a potential preventive therapy is to support YAP/TAZ signaling and to reduce dysfunctional epithelial cells at an early stage. However, despite little evidence, YAP/TAZ show prosenescent effects in specific circumstances [88–91]. The YAP-induced senescence might be a critical process for fibrosis, and further investigation is needed to better understand the pathogenesis of IPF.

## Conclusions

**Fig. 3:**
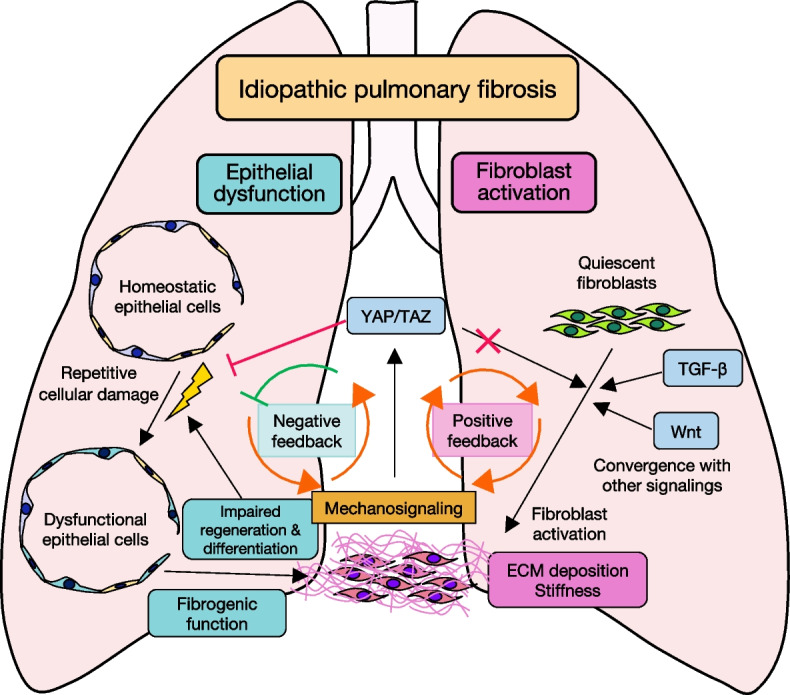
The conflicting roles of YAP/TAZ in the pathogenesis of IPF. Dysfunction of epithelial cells and activation of fibroblasts are the key components of IPF pathogenesis. Epithelial cell dysfunction is caused by repetitive damage due to various cellular stresses. The dysfunctional cells have impaired regeneration and differentiation, resulting in both dysregulation of homeostasis and fibrogenic effects. Meanwhile, the convergence of the Hippo, TGF-β, and Wnt signaling pathways affects the transcription of profibrotic molecules in fibroblasts, leading to excessive ECM deposition. Interestingly, YAP/TAZ mechanosignaling and ECM deposition (stiffness) form a positive feedback loop, representing the progressive feature of the pathogenesis of IPF. Conversely, YAP/TAZ maintain lung tissue homeostasis by counteracting the progression of IPF caused by epithelial dysfunction. The effects of YAP/TAZ on epithelial dysfunction and fibroblast activation appear to be in conflict; however, the timing of the effects might explain the discrepancy. In the early stage, YAP/TAZ can be assumed to prevent the pathogenesis of IPF by maintaining epithelial cell functions. When the negative feedback is ineffective, YAP/TAZ accelerate fibrosis by activating lung fibroblasts. Enhancing the homeostatic function in the early stage and inhibiting the profibrotic effect in the progressive state are promising therapeutic strategies

The key pathogenic mechanisms of IPF, a progressive fibrotic lung disease, are dysfunction of epithelial cells and activation of lung fibroblasts [2]. The Hippo pathway is a key signaling pathway for maintaining tissue homeostasis, and, when dysregulated, various diseases can develop [20]. In IPF, the Hippo pathway apparently has an opposite effect on the pathogenesis regarding epithelial cell dysfunction and fibroblast activation (Fig. 3). The effector signaling proteins YAP/TAZ act upon lung epithelial cells to maintain cellular functions, such as differentiation, regeneration, and cellular senescence. In contrast, the Hippo pathway converges with the TGF-β and Wnt signaling pathways and mechanotransduction and activates fibroblasts. With these actions, the Hippo pathway can be considered to play dual roles in IPF. The homeostatic and fibrogenic roles of YAP/TAZ might be to form a negative feedback loop for a healthy lung to evade the development of IPF. In other words, IPF might develop and accelerate when the negative feedback loop is ineffective. Furthermore, the Hippo pathway enhances the profibrotic stimuli by building a positive feedback loop with tissue stiffness and mechanosignaling, which can explain the progressive nature of IPF. Recent studies have shed light on the role of epithelial cells in accelerating the pathogenesis of IPF. In lungs with IPF, the normal alveolar architecture is destroyed and the distal airway, including alveoli, alveolar cysts, and ducts, is bronchiolized [92]. During such tissue remodeling, cell migration and airway epithelial cell proliferation are cooperatively regulated by YAP and signaling by mechanistic target of rapamycin kinase (mTOR)/phosphatidylinositol-3 kinase (PI3K)/alpha serine threonine-protein kinase (AKT) [31]. The mobilized state of epithelia activates fibroblasts and is maintained by the ERBB-YAP axis, implicating the fibrogenic role of the Hippo pathway in epithelial cells at an advanced stage of IPF pathogenesis [93].

The Hippo pathway is a potential therapeutic target of IPF, and several agents have been or are being developed. The main target has been the profibrotic effect of YAP/TAZ in fibroblasts; however, targeting the Hippo pathway to enhance its homeostatic effect at an early stage and to inhibit its fibrogenic effect at an advanced stage in epithelial cells might help prevent IPF from starting and advancing (see Additional file 1). Therefore, an efficient treatment strategy for IPF might be targeting the Hippo pathway via specific cell types and disease stages. A better understanding of the complex intercellular communications and convergence of the Hippo with other signaling pathways will help us discover new Hippo-targeted treatment strategies.

### Supplementary Information

**Additional file 1.** Summary figure. The main points of this review are summarized in the figure. The Hippo pathway opposingly affects the 2 key mechanisms of IPF pathogenesis: dysfunction of epithelial cells and activation of fibroblasts. Excessive ECM accumulation upregulates YAP/TAZ in fibroblasts via mechanosignaling, leading to further activation of fibroblasts and progressive feature of IPF. Supporting the YAP/TAZ activity in epithelial cells at an early stage and suppressing the YAP/TAZ activity in fibroblasts at an advanced stage can be a potential Hippo-targeted therapeutic strategy for IPF.

## Data Availability

Not applicable.

## Abbreviations

IPF: Idiopathic pulmonary fibrosis
ECM: Extracellular matrix
α-SMA: α Smooth muscle actin
TGF: Transforming growth factor
scRNA-seq: Single-cell RNA sequencing
GPCR: G protein–coupled receptor
STE20: Sterile 20
MST: Mammalian STE20-like protein kinase
LATS: Large tumor suppressor
YAP: Yes-associated protein
PSD-95: Postsynaptic density protein 95
DlgA: Drosophila disc large tumor suppressor
ZO-1: Zona occludens 1
PDZ: PSD-95, DlgA, and ZO-1
TAZ: Transcriptional coactivator with PDZ–binding motif
TEAD: Transcriptional enhancer associate domain
YAP/TAZ: YAP and TAZ
KRT5: Keratin 5
MMP7: Matrix metallopeptidase 7
EMT: Epithelial-mesenchymal transition
AT: Alveolar type
TP53: Tumor protein p53
NF: Nuclear factor
HIF1: Hypoxia-inducible factor 1
ALI: Acute lung injury
FZD: Frizzled
LRP: Low-density lipoprotein receptor-related protein
GSK: Glycogen synthase kinase
CK: Casein kinase
DVL: Disheveled
APC: Adenomatous polyposis coli
β-TrCP: β-Transducin repeat-containing protein
TCF: T-cell factor
Lef-1: Lymphoid enhancer-binding factor-1
LPA: Lysophosphatidic acid
S1P: Sphingosine-1-phosphate
CTGF: Connective-tissue growth factor
mtROS: Mitochondrial reactive oxygen species
IP: Prostacyclin
ROCK: Rho-kinase
NUAK1: NUAK family SNF1-like kinase 1
PAI-1: Plasminogen activator inhibitor type 1
DHX: Dihydrexidine
DRD1: Dopamine receptor D1
HMG-CoA: Hydroxymethylglutaryl-coenzyme A
miRNAs: MicroRNAs
EVs: Extracellular vesicles
mTOR: Mechanistic target of rapamycin kinase
PI3K: Phosphatidylinositol-3 kinase
AKT: Alpha serine threonine-protein kinase
