# Supplementary material for: Divergent roles of the Hippo pathway in the pathogenesis of idiopathic pulmonary fibrosis: tissue homeostasis and fibrosis
Source: Inflamm Regen. 2023 Sep 21;43:45. doi: 10.1186/s41232-023-00295-1 (PMC10512581; doi:10.1186/s41232-023-00295-1)
Supplement: Supplementary file 1 — Additional file 1. Summary figure. The main points of this review are summarized in the figure. The Hippo pathway opposingly affects the 2 key mechanisms of IPF pathogenesis: dysfunction of epithelial cells and activation of fibroblasts. Excessive ECM accumulation upregulates YAP/TAZ in fibroblasts via mechanosignaling, leading to further activation of fibroblasts and progressive feature of IPF. Supporting the YAP/TAZ activity in epithelial cells at an early stage and suppressing the YAP/TAZ activity in fibroblasts at an advanced stage can be a potential Hippo-targeted therapeutic strategy for IPF. [file 41232_2023_295_MOESM1_ESM.pdf]

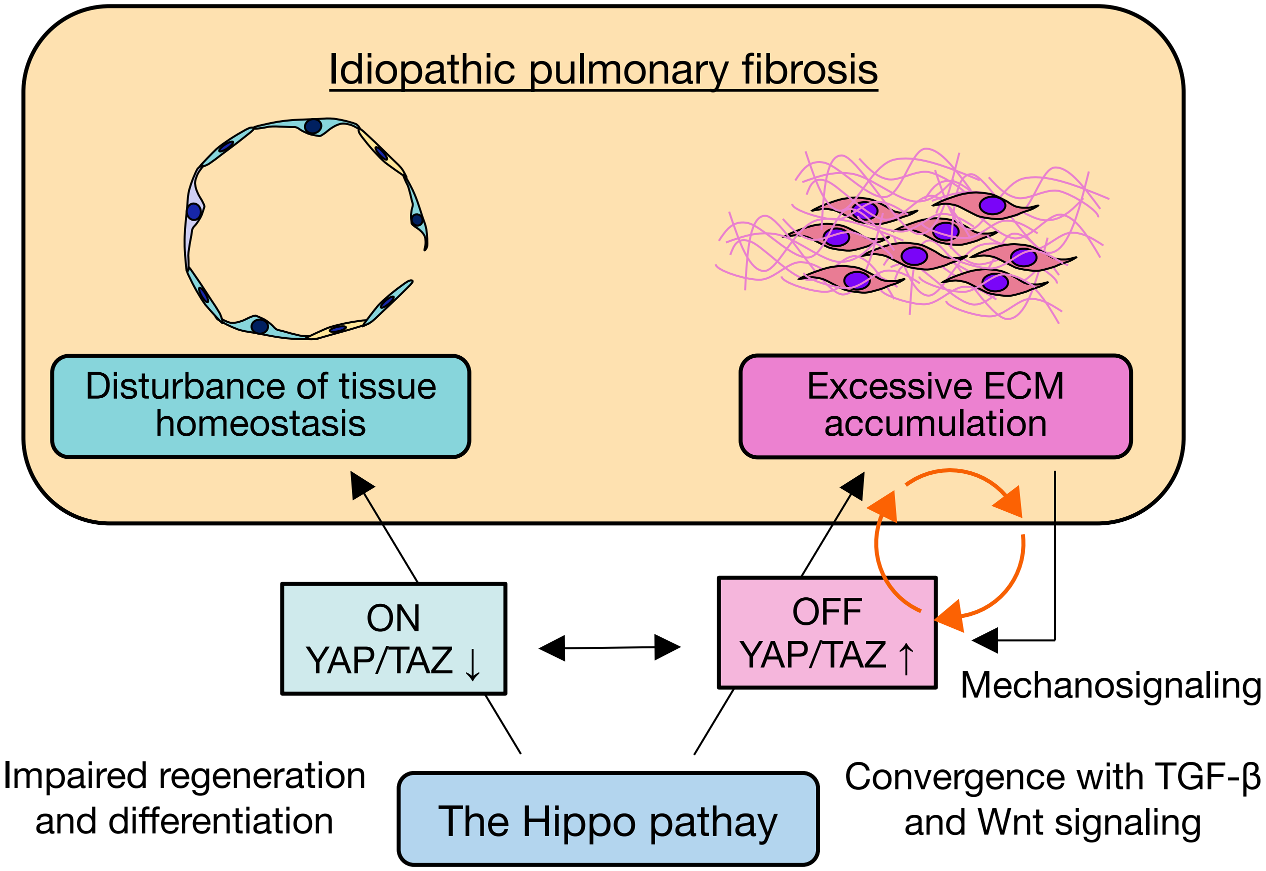

Proposed therapeutic strategies for IPF:

Early stage: support the YAP/TAZ activity in epithelial cells
Advanced stage: suppress the YAP/TAZ activity in fibroblasts
